# The Usefulness of Functional Near-Infrared Spectroscopy for the Assessment of Post-Stroke Depression

**DOI:** 10.3389/fnhum.2021.680847

**Published:** 2021-06-22

**Authors:** Masahiko Koyanagi, Mai Yamada, Toshio Higashi, Wataru Mitsunaga, Takefumi Moriuchi, Mitsuhiro Tsujihata

**Affiliations:** ^1^Department of Health Sciences, Nagasaki University Graduate School of Biomedical Sciences, Nagasaki, Japan; ^2^Department of Rehabilitation, Nagasaki Kita Hospital, Nagasaki, Japan; ^3^Department of Neurology, Nagasaki Kita Hospital, Nagasaki, Japan

**Keywords:** post-stroke depression, verbal fluency task, pre-frontal cortex, functional near-infrared spectroscopy, assessment

## Abstract

**Background:** Post-stroke depression (PSD) is the most common mood disorder following stroke and is also the main factor that limits the recovery and rehabilitation of patients with stroke. The prevalence of PSD is ~30%. Since there is no gold standard for the diagnosis and evaluation of PSD, it is important to raise awareness of PSD and to establish methods for its evaluation, early diagnosis, and treatment. In the field of psychiatry, functional near-infrared spectroscopy (fNIRS) has been used as a diagnostic tool for the measurement of oxygenated hemoglobin (oxy-Hb). This study aimed to assess whether fNIRS could be applied in the diagnosis and evaluation of PSD.

**Methods:** We recruited 45 patients with stroke, who were admitted to Nagasaki Kita Hospital between May 2015 and April 2019. The 17-item Hamilton Rating Scale for Depression (HAMD17), which is considered to be a useful screening and evaluation tool for PSD, was used for the assessment of patients after stroke; moreover, oxy-Hb was measured in the pre-frontal cortex. The subjects were divided into two groups: the depressed group (*n* = 13) and the non-depressed group (*n* = 32). We evaluated the correlation between the oxy-Hb integral values and HAMD17 scores.

**Results:** We investigated the relationship between the oxy-Hb integral values and HAMD17 total scores, and found a negative correlation between them (*ρ* = −0.331, *P* < 0.005). There was a significant difference in the oxy-Hb integral values during the activation task period between the depressed and non-depressed groups (3.16 ± 2.7 and 1.71 ± 2.4, respectively; *P* = 0.040). The results indicated that the patients of the depressed group showed lower oxy-Hb integral values and lower activation in the frontal lobe in comparison with the patients of the non-depressed group.

**Conclusion:** The present study highlights that the measurement of oxy-Hb by using fNIRS is a useful methodology for the diagnosis of PSD in patients after stroke.

## Introduction

Post-stroke depression (PSD) is the most frequent psychiatric problem and is strongly associated with a further worsening of physical and cognitive recoveries, functional outcomes, and quality of life (Paolucci, 2017; Shi et al., 2017). In addition, PSD is a serious problem for both stroke survivors and healthcare professionals, as it negatively affects the ability of the patient to engage in rehabilitation (Zhao et al., 2018). Recent meta-analyses and reviews have shown that the incidence of PSD ranges from 18 to 33%, and the prevalence of the post-stroke depressive disorder is 33.5% (Mitchell et al., 2017; Medeiros et al., 2020). Risk factors for PSD include genetic factors, age, sex, medical history, psychological history, type and severity of the stroke, location of lesions, degree of disability, and influence of social support (Ayerbe et al., 2013; Robinson and Jorge, 2016; Shi et al., 2017). However, there is currently no “gold standard” for the diagnosis and assessment of PSD due to differences in the timing of assessments, the use of different rating scales for depressive symptoms, and the associated signs and symptoms (e.g., aphasia and cognitive impairment) make the diagnosis and evaluation of PSD difficult (Laures-Gore et al., 2017; Zhao et al., 2018). The early recognition, prevention, and treatment of PSD are vital for the recovery and prognosis of stroke survivors.

Functional near-infrared spectroscopy (fNIRS) is a well-established, non-invasive tool that can be used to continuously assess regional tissue oxygenation at the bedside (Hong and Naseer, 2016). It has been used in different clinical settings, especially in the field of neuroscience (Obrig, 2014; Hong and Yaqub, 2019; Chen et al., 2020). The purpose of this study was to investigate whether fNIRS is useful for the assessment of PSD in patients with stroke.

## Materials and Methods

### Subjects

We recruited 45 patients with stroke (male, *n* = 32; female, *n* = 13; mean age, 67.8 ± 12.9 years), who were admitted to Nagasaki Kita Hospital from May 2015 to April 2019 (Table 1). Of the 45 patients, 26 had cerebral infarction and 19 had cerebral hemorrhage. The subjects were divided into two groups: the depressed group (*n* = 13) and the non-depressed group (*n* = 32) (Supplementary Table 1).

**Table 1:** Demographic and clinical data of the patients.

	**All patients**	**Depression group**	**Non-depression group**	**Group difference**
	**(*n* = 45)**	**(*n* = 13)**	**(*n* = 32)**	***p*-value**
Age^a^	67.8 ± 12.9	67.2 ± 9.6	67.8 ± 12.9	0.670
Gender, male/female^b^	32/13	9/4	23/9	0.859
Types of strokes^b^
Infarction/hemorrhage	26/19	6/7	20/12	0.321
Right hemisphere stroke/left hemisphere stroke	26/19	6/7	20/12	0.094
Time from onset to fNIRS (months)^a^	2.8 ± 1.3	3.0 ± 1.5	2.7 ± 1.2	0.548
MMSE (point)^a^	28 ± 2.2	28.2 ± 1.5	28.0 ± 2.4	0.678
FIM (score)^a^
Total	114.6 ± 11.0	108.4 ± 14.1	117.2 ±8.2	0.027^*^
Motor	82.8 ± 9.9	77.2 ± 13.1	85.0 ± 7.2	0.011^*^
Cognition	31.9 ± 3.7	31.2 ± 3.1	32.2 ± 3.8	0.122
FMA (point)^a^	50.2 ± 21.0	34.1 ± 23.9	56.7 ± 15.5	0.001^**^
BRS (stage)^a^
Upper limb	4.8 ± 1.3	3.7 ± 1.2	5.3 ± 0.9	<0.001^***^
Lower limb	5.0 ± 1.2	4.3 ± 1.2	5.3 ± 1.0	0.005^*^
Finger	4.9± 1.2	4.0 ± 1.2	5.3 ± 1.0	0.001^**^
HAMD17 (point)^a^	4.7 ± 3.9	9.8 ± 2.7	2.6 ± 1.9	<0.001^***^
oxy-Hb Integrated value^a^	2.7 ± 2.7	1.7 ± 2.4	3.2 ± 2.7	0.040^*^

*Values are mean ± SD*.

* *P < 0.05*,

** *P < 0.01*,

*** *P < 0.001*.

a *Mann–Whitney U-test*.

b *chi-squared test The Mann–Whitney U-test or Spearman rank correlation coefficient was used for comparing (these variables) each item between patients and controls. MMSE, Mini-Mental State Examinaton; FIM, Functional Independence Measure; FMA, Fugl-Meyer Assessment; BRS, Bruunstrom Recevery Stage; HAMD 17, The 17-item Hamilton Rating Scale for Depression*.

The inclusion criteria were as follows:

(1) Unilateral lesions of the cerebral hemispheres without involving the infratentorial region.

(2) More than 1 month after the onset of stroke.

(3) A Mini-Mental State Examination (MMSE) score of ≧ 24.

(4) Antipsychotic drug dose below the recommendation of the WHO.

(5) No complicating neurodegenerative diseases.

(6) No history of mental illness.

(7) Being able to sit for at least 30 min.

This study was approved by the Ethics Committee of Nagasaki University Graduate School of Biomedical Sciences (approval number: 17071374) and the Ethics Committee of Nagasaki Kita Hospital (approval number: 14–003). Written informed consent was obtained from all subjects in accordance with the Declaration of Helsinki.

### Assessment of PSD

The 17-item Hamilton Rating Scale for Depression (HAMD17), which is a comprehensive and quantitative measure of clinical symptoms of depression, was used to evaluate PSD (Hamilton, 1960; Meader et al., 2014). The severity of each item was scored on a scale of 0–2 or 0–4. The subjects who showed a severity score of ≤ 7 were classified into the non-depressive group. The patients of the depressed group were further classified according to their HAMD17 score, as follows: mild, 8–16 points; moderate, 17–23 points; and severe, ≥ 24 points. The HAMD17 was performed within 1 week of fNIRS measurement.

### Clinical Assessment of PSD

The following items that could affect the onset of PSD were examined:

- General information: age, sex, type of stroke, damaged hemisphere, date from the onset of stroke to the evaluation date, and medications.
- Cognitive function: MMSE.
- Activities of daily living: the sum of the functional independence measure (FIM) total score and exercise/cognitive items.
- Severity of paralysis after stroke: the upper limb function items of the Fugl-Meyer Assessment (FMA) and the upper limb, lower limb, and finger items of the Bruunstrom recovery stage (BRS).

### Measurement of fNIRS

#### Probe Positioning and Measurement Points

We used a 46-channel fNIRS instrument (OMM-3000/16, Shimadzu Corporation, Japan) to measure changes in the concentrations of oxygenated hemoglobin (oxy-Hb) and deoxyhemoglobin (deoxy-Hb) at three wavelengths (780, 805, and 830 nm) of infrared light based on the modified Beer-Lambert law (Maki et al., 1995; Yamashita et al., 1996).

The probes of the fNIRS machines were placed on the frontal and bilateral temporal regions of the subject. The frontal probes measured the hemoglobin concentration changes at 19 measurement points with the lowest probes positioned along the Fp1–Fp2 line according to the international 10/20 system used in electroencephalography (Okamoto et al., 2004; Zhu et al., 2018).

**Figure 1:**
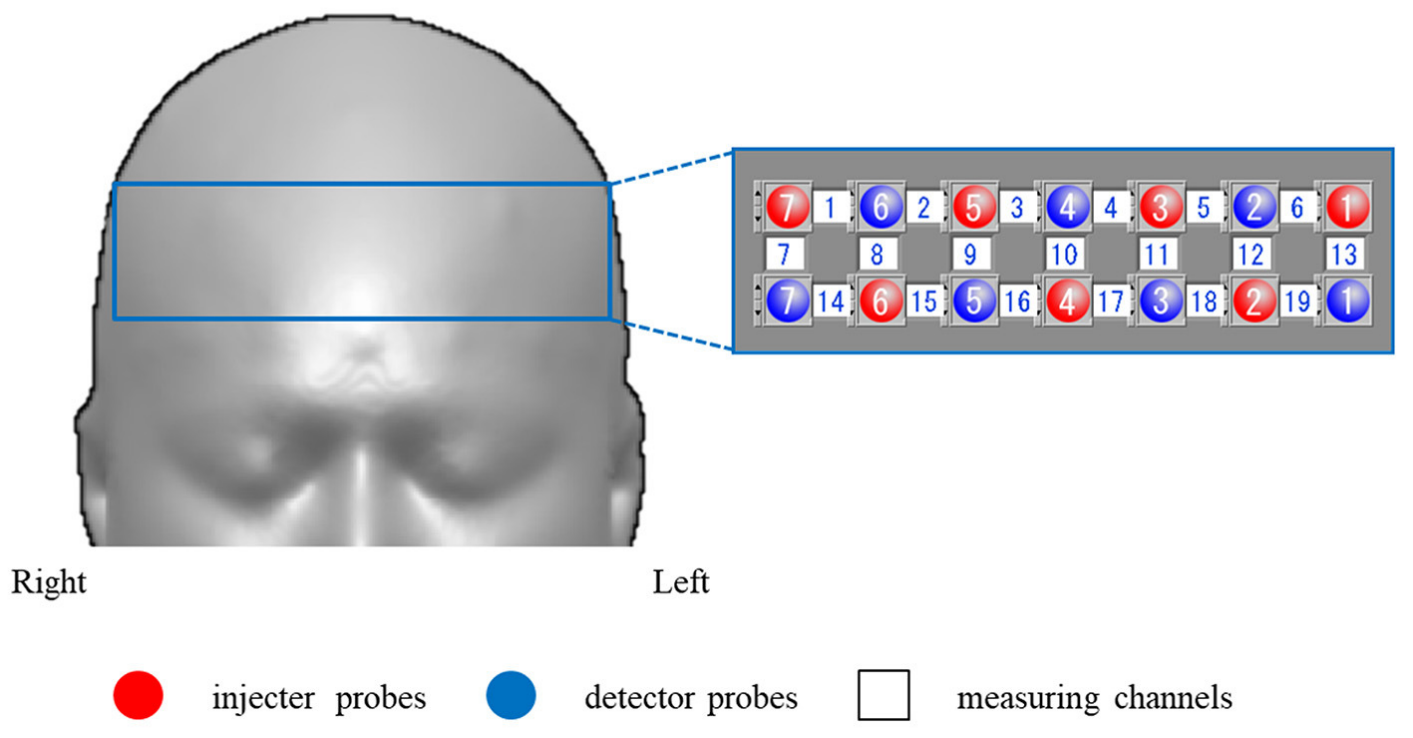
Functional near-infrared spectroscopy (fNIRS) measurement points. We used a 19-channel system with 14 optodes, seven injector probes, and seven detector probes solid white numbers denote the channels of measurement.

The distance between a detector probe and injector probe pair was set at 3 cm, and the area between the detector probe and injector probe pair was defined as a “channel” (Figure 1).

### Activation Task

**Figure 2:**
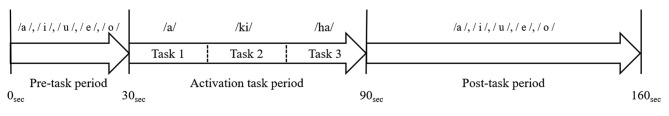
The verbal fluency task (VFT) protocol. The cognitive activation task had a block design and consisted of a 30-s pre-task baseline period, a 60-s VFT activation task period (three initial syllables, 20-s each), and a 70-s post-task baseline period.

Participants sat on a comfortable chair and were instructed to minimize any major body movements to avoid imaging artifacts, and the verbal fluency task (VFT) was used as an activation task (Takizawa et al., 2008, 2014). The VFT was a block design task and consisted of a 30-s pre-task baseline, a 60-s VFT task, and a 70-s post-task baseline. The subjects were instructed to generate as many words as possible where the initial syllable was /a/, /ki/, or /ha/. The three initial syllables changed every 20-s during the 60-s task. The subjects were also instructed to utter the Japanese syllables (/a /, / i /, / u /, / e /, and / o /) during the pre- and post-task baseline periods, which were used for baseline correction (Figure 2).

### Data Analysis of fNIRS

As no standardized method has been established for the analysis of fNIRS data, various approaches have been reported (Obrig and Villringer, 2003). In this study, after removing artifacts, the last 10 s of the 30-s pre-task period was used as the pre-task baseline and the first 55-s of the 70-s post-task period was used as the post-task baseline. Baseline correction was performed using the moving average method (Suto et al., 2004; Kameyama et al., 2006; Takizawa et al., 2008, 2014).

**Figure 3:**
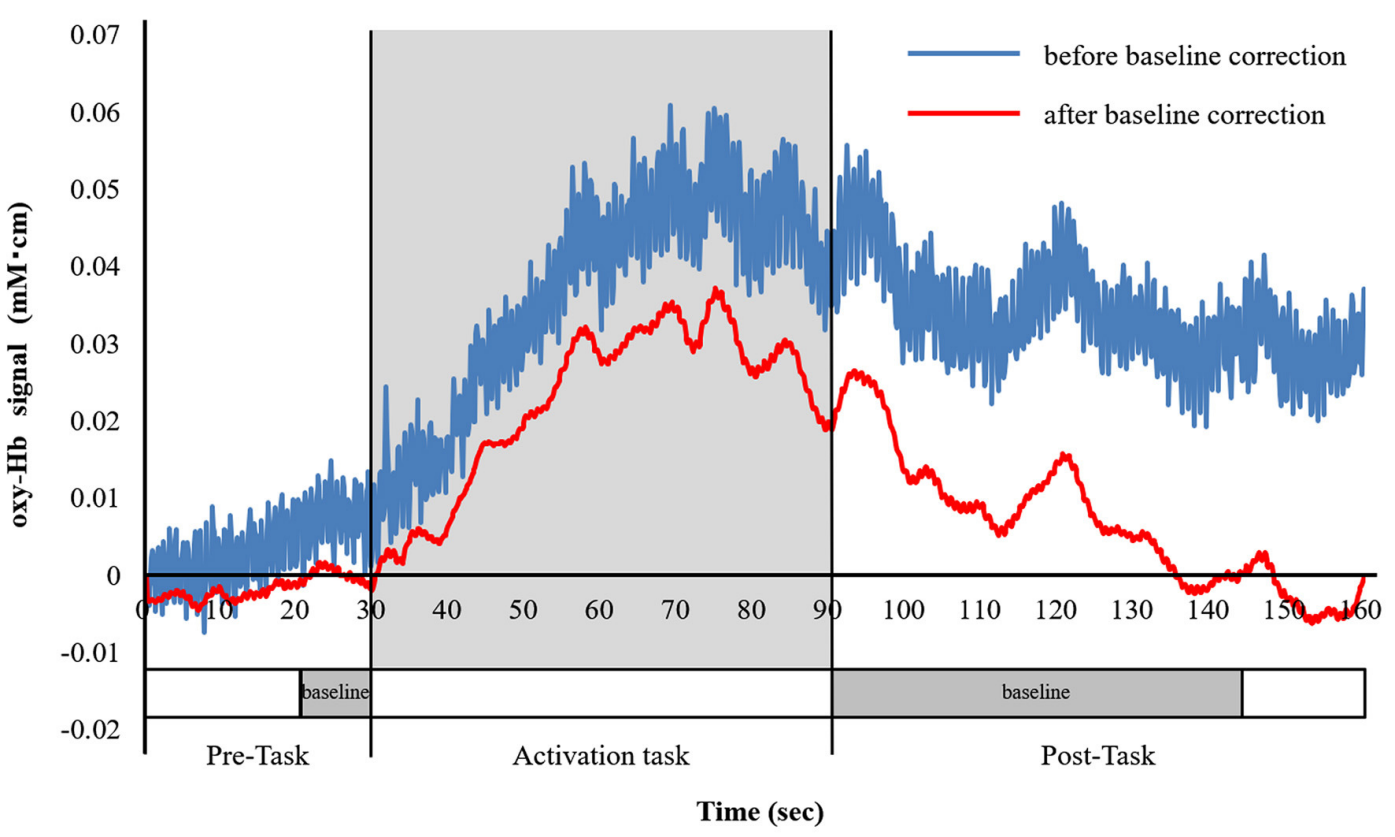
Analysis of the fNIRS data. The blue line indicates oxygenated hemoglobin (oxy-Hb) before the baseline correction, and the red line indicates oxy-Hb after the baseline correction. Baseline correction was performed in the last 10-s of the pre-task period and the first 55-s of the post-task period, which is shown by the dotted line on the time axis. Baseline correction was performed using the moving average method. We, then, averaged the oxy-Hb data of 19 channels and calculated the integral value of the task periods.

We obtained the integral value by averaging the data of the 19 channels, showed the size of the hemodynamic response during the activation task period (Figure 3).

Regarding the index of brain activity in fNIRS, oxy-Hb has been demonstrated to have a strong positive correlation with regional cerebral blood flow (Hoshi et al., 2001), and an increase in regional cerebral blood flow has been found to reflect an increase in neural activity (Jueptner and Weiller, 1995). Total hemoglobin roughly corresponds to blood flow variability but when the variability is small, it is unreliable (Hoshi et al., 2001; Miyai et al., 2001), and there are individual differences regarding changes in deoxy-Hb (Hesselmann et al., 2001). In this study, we only on focused oxy-Hb and analyzed the data (Takizawa et al., 2008, 2014).

### Experimental Environment and Position

The room was light- and sound-proofed to the best of authors' abilities. The instruments as well as the examiner were located behind the patient, where the examiner could examine the body movements of the patient without interfering with their visual field (Kondo et al., 2018). To prevent artifacts caused by visual stimuli, the personal computer screen was placed in front of the patient. In order to avoid physical movement artifacts and reduce the burden of fatigue and pain, the patient sat on a chair or wheelchair with a backrest, placed their hands on the desk, and placed their feet on the floor (Noda et al., 2012). fNIRS was performed after confirming general information.

### Statistical Analysis

Statistical analyses were performed using IBM SPSS for Macintosh, and statistical significance was set at *P* < 0.05. The relationship between the oxy-Hb integral value and the total score of HAMD17 was analyzed using Spearman's rank correlation coefficient. The Mann–Whitney U test was used to analyze age, time from onset to evaluation, MMSE, FIM, FMA, BRS, HAMD17, and oxy-Hb integral values. Sex and stroke types were examined using the chi-squared test. Spearman's rank correlation coefficient was used to analyze the correlation between the FIM, FMA, BRS, and HAMD17 total scores.

In statistical analysis, the Shapiro–Wilk test confirmed that all variables did not show a normal distribution.

## Results

### Clinical Data

**Figure 4:**
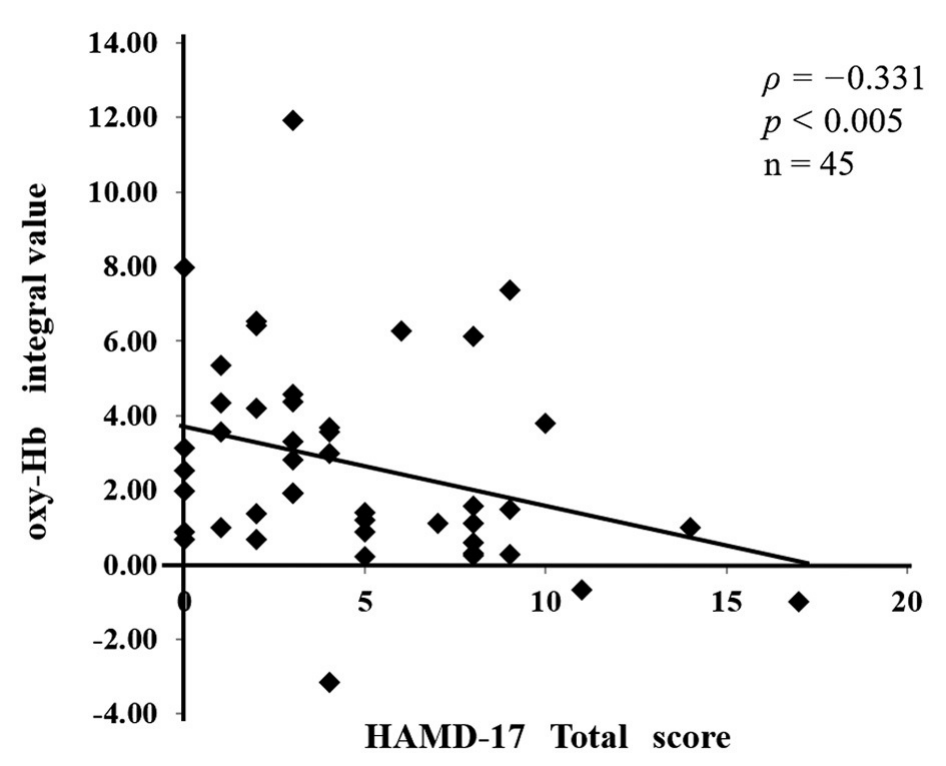
The relationship between the assessment of post-stroke depression (PSD) and the severity of paralysis. A negative correlation (ρ = −0.331, *P* < 0.005) was found between the oxy-Hb integral values and the total 17-item Hamilton Rating Scale for Depression (HAMD17) scores.

Table 1 shows the demographic characteristics of the 45 patients with stroke, who were divided into two groups: 32 patients without depression (the non-depressed group) and 13 patients with depression (the depressed group). We investigated the relationship between oxy-Hb integral values and HAMD17 total scores and found a negative correlation between them (ρ = −0.331, *P* < 0.005; Figure 4). Significant differences in the following items were observed between the non-depressed and depressed groups: FIM total score, *P* = 0.027; FIM motor items, *P* = 0.011; FMA, *P* = 0.001; BRS upper limb, *P* < 0.001; BRS lower limb, *P* = 0.005; BRS hand, *P* = 0.001; and HAMD17, *P* < 0.001. The following items did not differ between the two groups: age, sex, type of stroke, hemisphere, time from onset to assessment, and FIM cognitive items. The prevalence of PSD in the present study was 29%.

### Correlation Between the oxy-Hb Integral Value and the Severity of Paralysis

**Figure 5:**
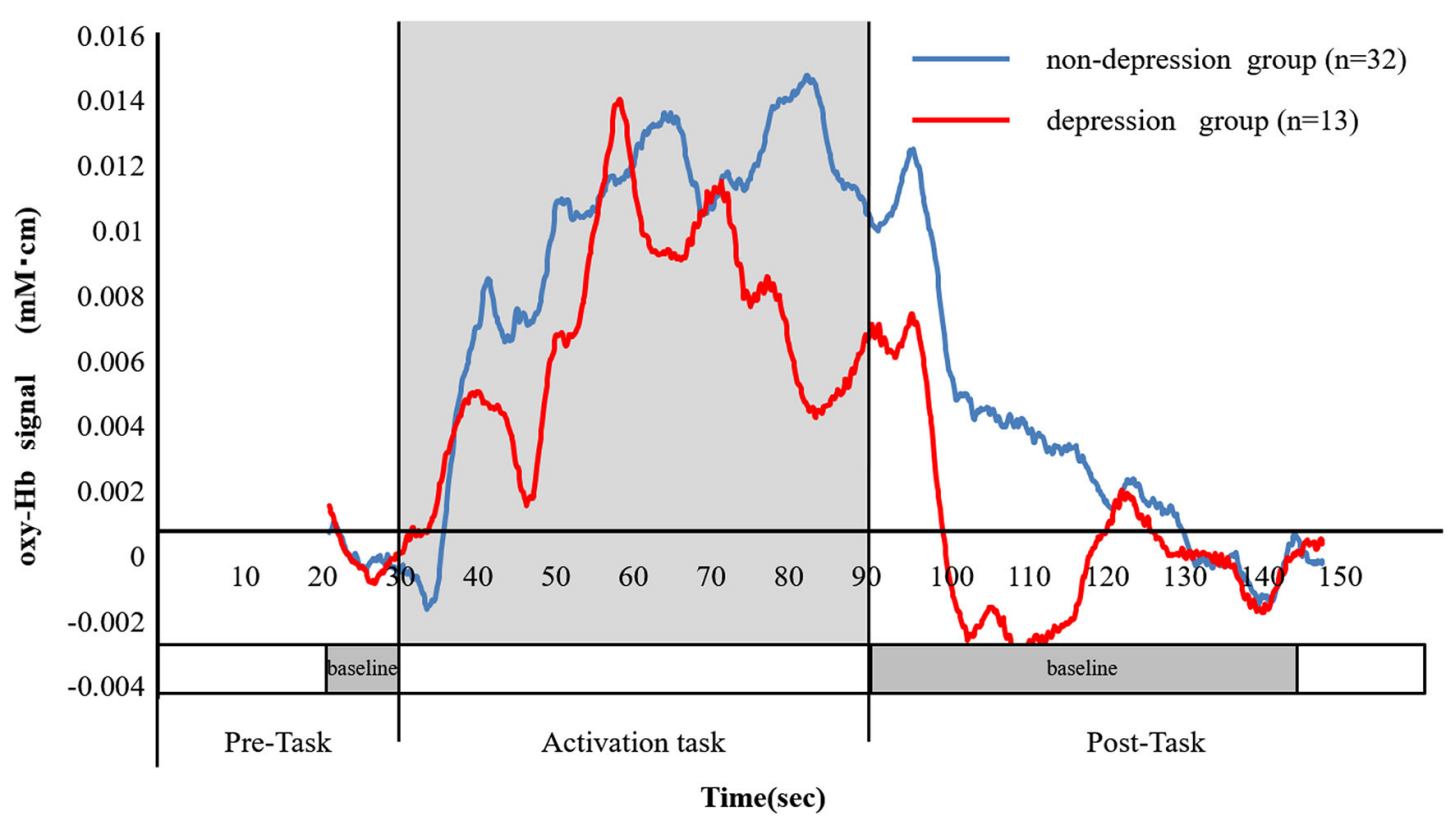
The change in the ratio of the oxy-Hb between the depressed and non-depressed groups. The blue line shows the mean oxy-Hb values in the non-depressed group (*n* = 32), while the red line shows the mean oxy-Hb values in the depressed group (*n* = 13). The mean of the oxy-Hb integral values in the non-depressed group was significantly higher than those of the depressed group (3.16 ± 2.7 and 1.71 ± 2.4, respectively, *P* = 0.040).

**Figure 6:**
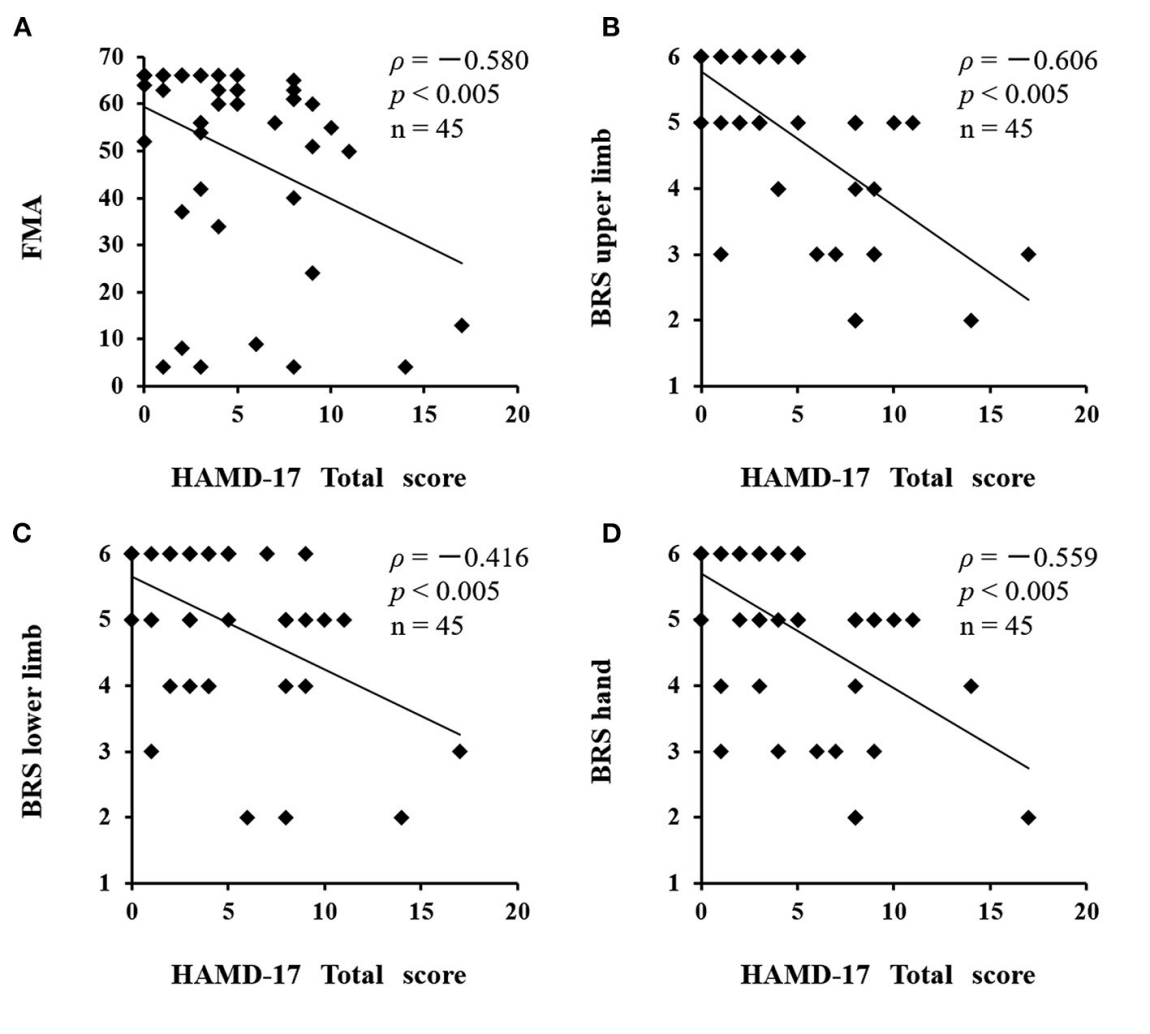
Correlation between HAMD17 total scores and severity of paralysis after stroke. A negative correlation was found between the total scores of the HAMD17 and the FMA and BRS values. **(A)** HAMD17: FMA, ρ = −0.580, *P* < 0.005; **(B)** HAMD17: BRS upper limb, ρ = −0.606, *P* < 0.005; **(C)** HAMD17: BRS lower limb, ρ = −0.416, *P* < 0.005; **(D)** HAMD17: BRS hand, ρ = −0.559, *P* < 0.005). HAMD17, The 17-item Hamilton Rating Scale for Depression; FMA, Fugl-Meyer Assessment; BRS, Bruunstrom Recovery Stage.

There was a significant difference between the depressed and non-depressed groups in the oxy-Hb integral value during the activation task period (3.16 ± 2.7 and 1.71 ± 2.4, respectively; *P* = 0.040). This result indicates that PSD may be induced by decreased oxy-Hb integral values and decreased activation in the frontal lobe (Figure 5). A negative correlation was found between the total scores of the HAMD17 and the FMA and BRS values (HAMD17: FMA, ρ = −0.580, *P* < 0.005; HAMD17: BRS upper limb, ρ = −0.606, *P* < 0.005; HAMD17: BRS lower limb, ρ = −0.416, *P* < 0.005; and HAMD17: BRS hand, ρ = −0.559, *P* < 0.005; Figure 6).

## Discussion

In this study, the subjects were classified into depressed and non-depressed groups based on the results of the HAMD17. A negative correlation was found between the oxy-Hb integral value and the HAMD17 total score (ρ = −0.331, *P* < 0.005). In addition, a significant difference was observed in the oxy-Hb integral values of the two groups (*P* = 0.040). The average value of the oxy-Hb integrated value during the activation task period was 1.71 ± 2.4 in the depressed group and 3.16 ± 2.7 in the non-depressed group, indicating that the depressed group had a lower oxy-Hb integral value and lower frontal lobe activation in comparison with the non-depressed group.

### Functional Near-Infrared Spectroscopy Study Using a VFT in PSD

There are no established diagnostic criteria or specific methods for the evaluation of PSD, and the pathophysiology of PSD has not yet been clarified (Ayerbe et al., 2013; Villa et al., 2018; Zhao et al., 2018). fNIRS is a well-established non-invasive tool that can be used to continuously assess regional tissue oxygenation at the bedside.

In this study, we investigated the frontal lobe functions in patients with PSD after stroke by using fNIRS, with a VFT as an activation task. We found a negative correlation between the fNIRS oxy-Hb integral value and the HAMD17 total score. We also found a significant difference in the oxy-Hb integral values of the non-depressed and depressed groups. Makizako et al. (2013) reported that oxygen-Hb values in the pre-frontal cortices of aged people (mean age, 76.1–6.7 years) were increased in comparison with baseline values: however, the effect of aging was not clarified. Although the non-depressed group showed a higher oxy-Hb integral value than the depressed group, we could not examine the effects of aging as healthy subjects were not included in this study.

To date, several papers have investigated major depression using fNIRS during activation tasks.

Noda (Noda et al., 2012) reported that the oxy-Hb increase in the frontal and right temporal cortex during a VFT was attenuated in patients with major depressive disorder (MDD) in comparison with healthy controls. The relationship between the severity of depression symptoms and the change in oxy-Hb was studied in 30 patients with MDD and 30 controls who were matched for age, sex, and intelligence quotient. The oxy-Hb increase during the task in patients was significantly smaller in comparison with controls. The mean increase in oxy-Hb, during the task, showed a significant negative correlation with the total score of the Hamilton Rating Scale for Depression 21-item version.

Zhang et al. (2015) reported similar results to those of Noda et al. (2012), where patients with MDD had significantly lower pre-frontal activation during cognitive tasks in comparison with healthy subjects.

Kawano (Kawano et al., 2016; Takamiya et al., 2017) investigated the relationship between oxy-Hb integral values and the severity of depression, as assessed using the Hamilton Depression Scale in patients with various psychiatric disorders, such as MDD, and found that the severity of depression was negatively correlated with the integral value in the frontal lobe, irrespective of psychiatric disorders.

In a situation where the pathophysiology of PSD is still unknown, we believe that the decrease in the integral value of oxy-Hb and the increase in HAMD17 in the frontal lobe of the depressed group show significant results, as they represent similar findings to previous studies in the field of psychiatry.

### Effects of Cerebral Lesions on PSD

Robinson et al. (1984) found that lesions involving the left frontal region of the brain were associated with a significantly higher frequency of depression during the first 2 months following acute stroke than comparable lesions of the right hemisphere or posterior lesions of the left hemisphere. Subsequently, the work of other investigators (Starkstein et al., 1987; Morris et al., 1996) identified that left-lateral frontal lobe, caudate, and putamen lesions were significantly more likely to produce depression during the acute stroke period than comparable lesions in the right hemisphere.

Nickel and Thomalla (2017) reviewed whether there is an association between PSD and stroke lesion characteristics, such as lesion size and lesion location. Available studies are hampered by methodological limitations, including the drawbacks of lesion analysis methods, small sample sizes, and the issue of patient selection. These limitations, together with differences in approaches to assess PSD and methods of image analysis, limit the comparability of results from different studies. Overall, the results are controversial, and no clear pattern of stroke lesions associated with PSD has emerged, although findings suggest that frontal stroke lesions are more so associated with a higher incidence of PSD. In the present study, the brain lesions in individual cases were diverse; thus, the relationship between the lesion and PSD could not be examined.

### Effects of Antipsychotic Drugs on fNIRS Signals

Anti-psychotropic medications have been reported to affect fNIRS signals. Among these medications (Schecklmann et al., 2008), high doses of antidepressants showed significant effects on NIRS signals in comparison with low doses. Three patients in this study took small amounts of antidepressants or antipsychotics; thus, we considered that the drugs had no effect on their fNIRS signals (Supplementary Table 1).

### Limitations

The NIRS methodology has several shortcomings. NIRS enables the measurement of hemoglobin concentration changes only as relative values, not as absolute values. Furthermore, NIRS has a relatively low spatial resolution in comparison with MRI, low cerebral penetration depth, and the contributions from extracerebral tissue, such as the skin and skull, may contaminate the NIRS signal. Due to a lack of standard quantification, the acquired hemoglobin data from various NIRS instruments are provided as relative values and are measured in different units (i.e., mmol mm, mmol/L, or arbitrary units). In this study, we obtained the integral value by averaging all the data of all channels, which showed the size of the hemodynamic response during the 60-s activation task period.

Regarding the NIRS reproducibility of NIRS data, the analysis of NIRS data requires careful interpretation analysis at the single subject and single-channel level is carefully interpreted (Schecklmann et al., 2008). In this study, we evaluated the analysis that was performed using the average values of multiple channels.

In recent years, some studies have been conducted using new analysis methods such as machine learning, and it is necessary to further study the methods of analysis (Kang and Cho, 2020).

Further studies, with larger study populations, are necessary to investigate the relationship between the lesion and PSD.

### Conclusions

We investigated the frontal lobe functions in patients with PSD after stroke by using fNIRS with VFT as an activation task. We found a negative correlation between the fNIRS oxy-Hb integral value and the HAMD17 total score, as well as a significant difference in the oxy-Hb integral value between the non-depressed and depressed groups. Currently, there is no “gold standard” for the diagnosis and assessment of PSD. The present study indicates that the measurement of oxy-Hb using fNIRS is a useful diagnostic method for PSD in patients after stroke.

## Data Availability Statement

The original contributions presented in the study are included in the article Supplementary Material, further inquiries can be directed to the corresponding author/s.

## Ethics Statement

The studies involving human participants were reviewed and approved by Ethics Committee of Nagasaki University Graduate School of Biomedical Sciences. The patients/participants provided their written informed consent to participate in this study.

## Author Contributions

MK and MY are occupational therapists who were involved in PSD rehabilitation treatment and performed the experiments. MK, MY, WM, TM, and TH analyzed the data. MT was involved as a specialist from the Department of Neurology. MT, MY, and TH contributed to the preparation of the manuscript. All authors agreed with the items listed in the submission manual.

## Conflict of Interest

The authors declare that the research was conducted in the absence of any commercial or financial relationships that could be construed as a potential conflict of interest.

## Abbreviations

PSD: post-stroke depression
VFT: verbal fluency task
fNIRS: functional near-infrared spectroscopy
HAMD17: 17-item Hamilton Rating Scale for Depression
oxy-Hb: oxygenated hemoglobin
FIM: functional independence measure
FMA: Fugl-Meyer Assessment
BRS: Bruunstrom Recovery Stage
MDD: major depressive disorder.

## References

[B1] Ayerbe L. Ayis S. Wolfe C. D. Rudd A. G. (2013). Natural history, predictors and outcomes of depression after stroke: systematic review and meta-analysis. Br. J. Psychiatry 202, 14–21. 10.1192/bjp.bp.111.107664 23284148

[B2] Chen W. L. Wagner J. Heugel N. Sugar J. Lee Y. W. Conant L.. (2020). Functional near-infrared spectroscopy and its clinical application in the field of neuroscience: advances and future directions. Front. Neurosci. 14:724. 10.3389/fnins.2020.00724 32742257 PMC7364176

[B3] Hamilton M. (1960). A rating scale for depression. J. Neurol. Neurosurg. Psychiatry 23, 56–62. 10.1136/jnnp.23.1.56 14399272 PMC495331

[B4] Hesselmann V. Zaro Weber O. Wedekind C. Krings T. Schulte O. Kugel H.. (2001). Age related signal decrease in functional magnetic resonance imaging during motor stimulation in humans. Neurosci. Lett. 308, 141–144. 10.1016/S0304-3940(01)01920-6 11479008

[B5] Hong K. S. Naseer N. (2016). Reduction of delay in detecting initial dips from functional near-infrared spectroscopy signals using vector-based phase analysis. Int. J. Neural Syst. 26, 1650012. 10.1142/S012906571650012X 26971785

[B6] Hong K. S. Yaqub M. A. (2019). Application of functional near-infrared spectroscopy in the healthcare industry: a review. J. Innov. Opt. Health Sci. 12:930012. 10.1142/S179354581930012X

[B7] Hoshi Y. Kobayashi N. Tamura M. (2001). Interpretation of near-infrared spectroscopy signals: a study with a newly developed perfused rat brain model. J. Appl. Physiol. 90, 1657–1662. 10.1152/jappl.2001.90.5.1657 11299252

[B8] Jueptner M. Weiller C. (1995). Review: does measurement of regional cerebral blood flow reflect synaptic activity? Implications for PET and fMRI. Neuroimage 2, 148–156. 10.1006/nimg.1995.1017 9343597

[B9] Kameyama M. Fukuda M. Yamagishi Y. Sato T. Uehara T. Ito M.. (2006). Frontal lobe function in bipolar disorder: a multichannel near-infrared spectroscopy study. Neuroimage 29, 172–184. 10.1016/j.neuroimage.2005.07.025 16125979

[B10] Kang S. G. Cho S. E. (2020). Neuroimaging biomarkers for predicting treatment response and recurrence of major depressive disorder. Int. J. Mol.Sci. 20:2148. 10.3390/ijms21062148 32245086 PMC7139562

[B11] Kawano M. Kanazawa T. Kikuyama H. Tsutsumi A. Kinoshita S. Kawabata Y.. (2016). Correlation between frontal lobe oxy-hemoglobin and severity of depression assessed using near-infrared spectroscopy. J. Affect. Disord. 205, 154–158. 10.1016/j.jad.2016.07.013 27449547

[B12] Kondo A. Shoji Y. Morita K. Sato M. Ishii Y. Yanagimoto H.. (2018). Characteristics of oxygenated hemoglobin concentration change during pleasant and unpleasant image-recall tasks in patients with depression: comparison with healthy subjects. Psychiatry Clin. Neurosci. 72, 611–622. 10.1111/pcn.12684 29808572

[B13] Laures-Gore J. S. Farina M. Moore E. Russell S. (2017). Stress and depression scales in aphasia: relation between the aphasia depression rating scale, stroke aphasia depression questionnaire-10, and perceived stress scale. Top. Stroke Rehabil. 24, 114–118. 10.1080/10749357.2016.1198528 27348232 PMC5376271

[B14] Maki A. Yamashita Y. Ito Y. Watanabe E. Mayanagi Y. Koizumi H. (1995). Spatial and temporal analysis of human motor activity using noninvasive NIR topography. Med. Phys. 22, 1997–2005. 10.1118/1.597496 8746704

[B15] Makizako H. Doi T. Shimada H. Park H. Uemura K. Yoshida D.. (2013). Relationship between going outdoors daily and activation of the prefrontal cortex during verbal fluency tasks (VFTs) among older adults: a near-infrared spectroscopy study. Arch. Gerontol. Geriatr. 56, 118–123. 10.1016/j.archger.2012.08.017 22995341

[B16] Meader N. Moe-Byrne T. Llewellyn A. Mitchell A. J. (2014). Screening for post-stroke major depression: a meta-analysis of diagnostic validity studies. J. Neurol. Neurosurg. Psychiatry 85, 198–206. 10.1136/jnnp-2012-304194 23385849

[B17] Medeiros G. C. Roy D. Kontos N. Beach S. R. (2020). Post-stroke depression: a 2020 updated review. Gen. Hosp. Psychiatry 66,70–80. 10.1016/j.genhosppsych.2020.06.011 32717644

[B18] Mitchell A. J. Sheth B. Gill J. Yadegarfar M. Stubbs B. Yadegarfar M.. (2017). Prevalence and predictors of post-stroke mood disorders: a meta-analysis and meta-regression of depression, anxiety, and adjustment disorder. Gen. Hosp. Psychiatry 47, 48–60. 10.1016/j.genhosppsych.2017.04.001 28807138

[B19] Miyai I. Tanabe H. C. Sase I. Eda H. Oda I. Konishi I.. (2001). Cortical mapping of gait in humans: a near-infrared spectroscopic topography study. Neuroimage 14, 1186–1192. 10.1006/nimg.2001.0905 11697950

[B20] Morris P. L. Robinson R. G. de Carvalho M. L. Albert P. Wells J. C. Samuels J. F.. (1996). Lesion characteristics and depressed mood in stroke data bank study. J. Neuropsychiatr. Clin. Neurosci. 8, 153–159. 10.1176/jnp.8.2.153 9081550

[B21] Nickel A. Thomalla G. (2017). Post-stroke depression: impact of lesion location and methodological limitations: a topical review. Front. Neurol. 8:498. 10.3389/fneur.2017.00498 28983281 PMC5613107

[B22] Noda T. Yoshida S. Matsuda T. Okamoto N. Sakamoto K. Koseki S.. (2012). Frontal and right temporal activations correlate negatively with depression severity during verbal fluency tasks: a multi-channel near-infrared spectroscopy study. J. Psychiatr. Res. 46, 905–912. 10.1016/j.jpsychires.2012.04.001 22572569

[B23] Obrig H. (2014). NIRS in clinical neurology - a “promising” tool? Neuroimage 85, 535–546. 10.1016/j.neuroimage.2013.03.045 23558099

[B24] Obrig H. Villringer A. (2003). Beyond visible light imaging of the human brain. J. Cereb. Blood Flow Metab. 23, 1–18. 10.1097/01.WCB.0000043472.45775.29 12500086

[B25] Okamoto M. Dan H. Sakamoto K. Takeo K. Shimizu K. Kohno S.. (2004). Three-dimensional probabilistic anatomical craniocerebral correlation via the international 10–20 system oriented for transcranial functional brain mapping. Neuroimage 21, 99–111. 10.1016/j.neuroimage.2003.08.026 14741647

[B26] Paolucci S. (2017). Advances in antidepressants for treating post-stroke depression. Expert Opin. Pharmacother. 18, 1011–1017. 10.1080/14656566.2017.1334765 28535081

[B27] Robinson R. G. Jorge R. E. (2016). Post-stroke depression: a review. Am. J. Psychiatry 173, 221–231. 10.1176/appi.ajp.2015.15030363 26684921

[B28] Robinson R. G. Kubos K. L. Starr L. B. Rao K. Price T. R. (1984). Mood disorders in stroke patients. Importance of location of lesion. Brain 107, 81–93. 10.1093/brain/107.1.81 6697163

[B29] Schecklmann M. Ehlis A. C. Plichta M. M. Fallgatter A. J. (2008). Functional near-infrared spectroscopy: a long-term reliable tool for measuring brain activity during verbal fluency. Neuroimage 43, 147–155. 10.1016/j.neuroimage.2008.06.032 18657624

[B30] Shi Y. Yang D. Zeng Y. Wu W. (2017). Risk factors for post-stroke depression: a meta-analysis. Front. Aging Neurosci. 9:218. 10.3389/fnagi.2017.00218-28744213 PMC5504146

[B31] Starkstein S. E. Robinson R. G. Price T. R. (1987). Comparison of cortical and subcortical lesions in the production of poststroke mood disorders. Brain 110, 1045–1059. 10.1093/brain/110.4.1045 3651794

[B32] Suto T. Fukuda M. Ito M. Uehara T. Mikuni M. (2004). Multichannel near-infrared spectroscopy in depression and schizophrenia: cognitive brain activation study. Biol. Psychiatry 55, 501–511. 10.1016/j.biopsych.2003.09.008 15023578

[B33] Takamiya A. Hirano J. Ebuchi Y. Ogino S. Shimegi K. Emura H.. (2017). High-dose antidepressants affect near-infrared spectroscopy signals: a retrospective study. NeuroImage Clin. 14, 648–655. 10.1016/j.nicl.2017.02.008 28348956 PMC5357702

[B34] Takizawa R. Fukuda M. Kawasaki S. Kasai K. Mimura M. Pu S.. (2014). Joint project for psychiatric application of near-infrared spectroscopy (JPSY-NIRS) group. Neuroimaging-aided differential diagnosis of the depressive state. NeuroImage 85, 498–507. 10.1016/j.neuroimage.2013.05.126 23764293

[B35] Takizawa R. Kasai K. Kawakubo Y. Marumo K. Kawasaki S. Yamasue H.. (2008). Reduced frontopolar activation during verbal fluency task in schizophrenia: a multi-channel near-infrared spectroscopy study. Schizophr. Res. 99, 250–262. 10.1016/j.schres.2007.10.025 18063344

[B36] Villa R. F. Ferrari F. Moretti A. (2018). Post-stroke depression: mechanisms and pharmacological treatment. Pharmacol. Ther. 184, 131–144. 10.1016/j.pharmthera.2017.11.005 29128343

[B37] Yamashita Y. Maki A. Ito Y. Watanabe E. Mayanagi Y. Koizumi H. (1996). Noninvasive near-infrared topography of human brain activity using intensity modulation spectroscopy. Opt. Eng. 35, 1046–1049. 10.1117/1.600721

[B38] Zhang H. Dong W. Dang W. Quan W. Tian J. Chen R.. (2015). Near-infrared spectroscopy for examination of prefrontal activation during cognitive tasks in patients with major depressive disorder: a meta-analysis of observational studies. Psychiatry Clin. Neurosci. 69, 22–33. 10.1111/pcn.12209 24897940

[B39] Zhao F. Y. Yue Y. Y. Li L. Lang S. Y. Wang M. W. Du X. D.. (2018). Clinical practice guidelines for post-stroke depression in China. Braz. J. Psychiatry 40, 325–334. 10.1590/1516-4446-2017-2343 29412338 PMC6899404

[B40] Zhu Y. Quan W. Wang H. Ma Y. Yan J. Zhang H.. (2018). Prefrontal activation during a working memory task differs between patients with unipolar and bipolar depression: a preliminary exploratory study. J. Affect. Disord. 225, 64–70. 10.1016/j.jad.2017.07.031 28797920

